# Soluplus^®^-Based Pharmaceutical Formulations: Recent Advances in Drug Delivery and Biomedical Applications

**DOI:** 10.3390/ijms26041499

**Published:** 2025-02-11

**Authors:** Nerea Guembe-Michel, Paul Nguewa, Gustavo González-Gaitano

**Affiliations:** 1 Department of Chemistry, School of Science, University of Navarra, 31080 Pamplona, Spain; nguembe.1@alumni.unav.es; 2 Department of Microbiology and Parasitology, Navarra Institute for Health Research (IdisNA), University of Navarra, 31080 Pamplona, Spain

**Keywords:** Soluplus, drug delivery, polymeric micelles, solid dispersion, hot-melt extrusion, spray drying, electrospinning, drug–polymer layering

## Abstract

Poor water solubility remains a significant challenge in the pharmaceutical industry that limits the therapeutic efficacy and bioavailability of many active pharmaceuticals. Soluplus^®^ (SLP), an amphiphilic graft copolymer made of polyethylene glycol, polyvinyl caprolactam, and polyvinyl acetate, has been gaining interest in recent years as it addresses these limitations by acting as a versatile carrier. Its ability to form stable amorphous dispersions and enhance drug solubility, as well as its physicochemical properties, support its role as a key excipient in advanced drug delivery systems. Recent investigations have demonstrated the adaptability of SLP in addressing drug delivery requirements, offering controlled release, improved targeting, and superior therapeutic outcomes. This review examines some key formulation methods that make use of SLP, including hot-melt extrusion, spray drying, electrospinning, drug–polymer layering, and capsule and tablet formulations, highlighting the capacity of SLP to overcome formulation challenges. Biomedical applications of SLP have also been explored, with a focus on its role in improving the delivery of antitumoral, anti-inflammatory, antimicrobial, and antiparasitic drugs.

## 1. Introduction

Some orally administered drugs remain unabsorbed, limiting their therapeutic options and their clinical efficacy [1,2]. It has been observed that a set of medicines designed to treat chronic conditions or to manage severe illnesses fail to achieve their full potential because a great number of them exhibit poor solubility in water, which is a critical factor for their effective absorption [3]. As a result, only a small fraction of the administered dose of active principle reaches the systemic circulation and, therefore, the target site. These challenges are widespread, affecting a wide category of pharmaceutical compounds, particularly class-II and class-IV drugs, according to the Biopharmaceutics Classification System (BCS).

Poor water solubility has more implications than just inefficacy, as it can also lead to different therapeutic and side effects. BCS class-II drugs have high permeability and can easily cross biological membranes and consequently, their absorptions largely depend on their dissolution rates in the gastrointestinal fluids. A well-known example is ibuprofen, widely used as a painkiller and anti-inflammatory, which, despite its high permeability, often demonstrates variable absorption levels because of its limited solubility [4,5]. Similarly, when dealing with class-IV drugs that display both poor solubility and permeability, additional challenges are faced in order to achieve therapeutic effectiveness, such as complex matrices or the incompatibility of formulating high-dose drugs [6]. The combination of these factors highlights the need for innovative approaches to improve the pharmacokinetic profiles of these drugs, ensuring their efficient absorption that can permit exerting the intended therapeutic effects.

Different strategies to target key factors in drug formulation and delivery have been developed in order to enhance their solubility and absorption, thereby improving their bioavailability [7]. Examples include the production of formulations in which a drug is dispersed in a solid matrix, as well as lipid-based formulations, capable of enhancing the solubility of lipophilic drugs by the formation of oil-in-water emulsions upon contact with gastrointestinal fluids. The use of nanosuspensions is another approach that is attracting attention lately, in which a drug is dispersed in a colloidal nanoscale system, significantly increasing its surface area and, consequently, its dissolution rate [8,9].

In recent years, polymer-based drug delivery systems have gained ground because of their ability to modulate the release profile of drugs in a more controlled and sustained manner. This is particularly beneficial for drugs that require precise dosages to avoid adverse effects. Polymers classified as “generally recognized as safe” (GRAS), such as polyvinylpyrrolidone (PVP), polyethylene glycol (PEG), low molecular weight chitosan, and certain amphiphilic block copolymers, are commonly employed because of their biocompatibility and capacity to interact with a wide range of drugs [10,11]. For example, PVP has been effectively used via solid dispersion techniques to improve the dissolution rate of itraconazole, an antifungal agent, resulting in better therapeutic outcomes [10]. Similarly, López-Rios de Castro et al. (2024) concluded that PEG-PLGA polymeric nanoparticles were able to solubilize therapeutic peptides [12]. Likewise, poloxamers (better known by their trade name, Pluronic^®^, from BASF, Tarragona, Spain), a family of triblock copolymers constituted of polyethylene oxide (PEO) and polypropylene oxide (PPO) blocks, have been widely utilized to enhance the solubility of drugs such as paclitaxel, an anti-cancer agent with very limited water solubility [10]. Poloxamines (Tetronic^®^, also from BASF, Tarragona, Spain) are another family of amphiphiles, formed by four arms of PEO and PPO blocks connected by an ethylene diamine spacer, which confers pH-sensitivity to the polymer, as an advantage over their linear counterparts, Pluronic^®^. They have been used recently in the form of micelles and gels [13,14] and in combination with polymeric nanofibers [15] for the delivery of miltefosine, an antiparasitic drug used for the treatment of leishmaniasis.

Among amphiphilic block copolymers, Soluplus^®^ (SLP, hereafter), an amphiphilic water-soluble graft copolymer developed by BASF, stands out as particularly effective in addressing the solubility challenges posed by poorly water-soluble drugs as well as a matrix polymer for solid solutions [16]. Until now, most articles dealing with SLP have focused on the features and applications of this copolymer in drug delivery, highlighting its advantages, drug incorporation methods, and the physicochemical characteristics of SLP-based formulations. In this review, we have focused on the biomedical applications of this polymer. This review is structured to explore the physicochemical properties of SLP, followed by a discussion of potential formulation methods, and concludes with some of its relevant biomedical applications.

## 2. Physicochemical Features of SLP

**Figure 1:**
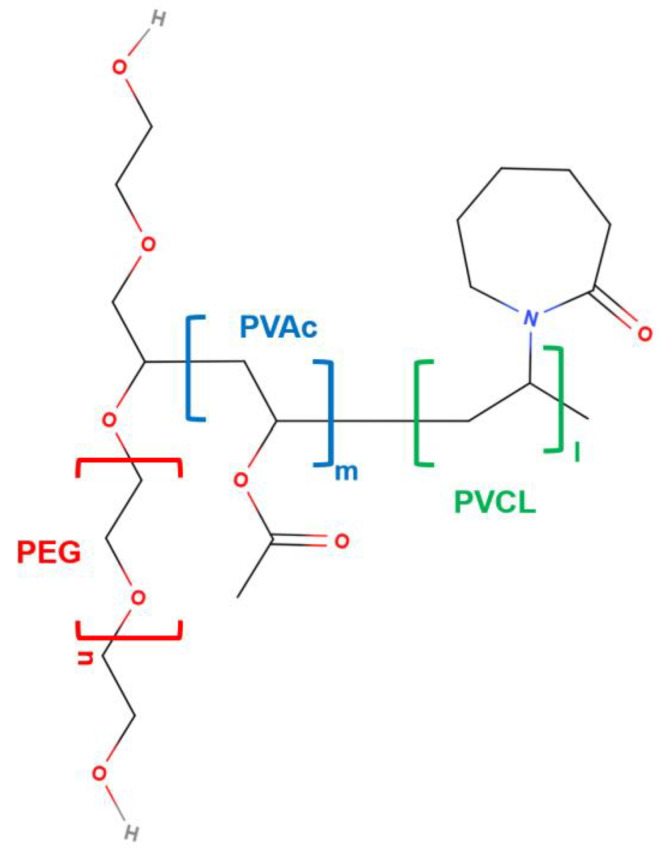
Molecular structure of SLP, showing the block structure. Red: polyethylene glycol (PEG, 13%); green: polyvinyl caprolactam (PVCL, 57%); blue: polyvinyl acetate (PVAc, 30%).

From the structural viewpoint, SLP is an amphiphilic tri-block copolymer (Figure 1). The hydrophilic part of SLP consists of a polyethylene glycol (PEG) block, which represents 13% of the polymer mass, while the lipophilic part is formed by polyvinyl caprolactam, PVCL (57%), and polyvinyl acetate, PVAc (30%), with an average molar mass of 118,000 g/mol [16]. When used above its critical micelle concentration (CMC, 7.6 mg/L at 23 °C), SLP can form micelles that encapsulate poorly soluble drugs, improving their dissolution and absorption. Below the CMC, it acts as a stabilizing agent, preventing precipitation by inhibiting nucleation and crystal growth and maintaining the drugs in a supersaturated state [2]. The micelles are relatively large, with typical diameters ranging from 70 to 100 nm at pH 7 [16].

SLP can also be solubilized in acetone (up to 50%), methanol (up to 45%), ethanol (up to 25%), dimethylformamide (up to 50%), and in 1:1 mixtures of methanol/acetone (up to 50%) and ethanol/acetone (up to 45%). At higher concentrations, the solutions become turbid because of the formation of colloidal clusters of micelles. According to the literature, SLP granulates measured by laser diffraction have a diameter of 340 µm, and the glass transition temperature is around 70 °C [16].

The self-aggregation of SLP in water has been investigated extensively by nuclear magnetic resonance (NMR), small angle X-ray scattering (SAXS), light scattering methods, and viscosity measurements [17]. SLP unimers form core–shell micelles above 10^−3^ g/mL (CMC), a structure that remains constant up to 0.2 g/mL. They also reported that the majority of the molecules were arranged in small structures formed by a few unimers (hydrodynamic radius, R_h_ = 3 nm). Some of these small structures were self-assembled into larger objects, forming micelles (R_h_ = 35 nm, at room temperature), thus concluding that there would be a coexistence between polymer coils and micellar structures [17].

In a comprehensive approach to studying the structure of SLP aggregates, Sofroniou et al. (2022) used small angle neutron scattering (SANS) combined with differential scanning calorimetry (DSC), NMR, and rheometry methods [18]. They found that SLP micelles could be modeled as spherical particles in the range of 1–15%, with a diffuse boundary and an average radius of approximately 22 nm, by considering a 2-Yukawa potential. The shell of these aggregates is highly hydrated, with a significant penetration of solvent into the micelle core. In contrast, the SANS patterns for 20–55% SLP solutions were best described by the Teubner–Strey model for bicontinuous structures, suggesting a well-organized arrangement of micelles.

**Figure 2:**
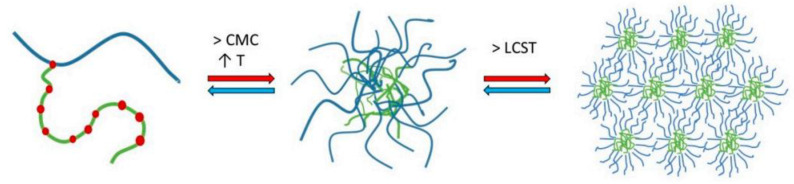
Representation of the micellization mechanism for SLP in aqueous solutions. From Alopaeus et al., with permission [19].

Alopaeus et al. (2019) have reported on the effect of pH, ion content and type on the micellization of SLP [19], results that are consistent with the prior findings by Wu et al. (2017) [20]. They also described that micellization occurs in three stages. First, micelles start forming when both CMC and CMT (critical micelle temperature) are surpassed. Then, as the temperature continues to increase, the PEG chains of SLP dehydrate, clustering below the lower critical solution temperature (LCST). Finally, once the LCST is exceeded, micelles undergo gelation (Figure 2) [19,20].

SLP sols exhibit a colloidal, light blue appearance, highly flowable at 25 °C [20]. However, at 37 °C, the sols undergo crosslinking to form opaque hydrogels, driven by hydrophobic interactions occurring between chain segments and the disruption of hydrogen bonds between water and PEGylated chains. The sol-gel transition in these hydrogels follows a temperature-induced shift from a liquid-like sol to an elastic, gel-like state. SLP concentration, ion types, ionic strength, and pH are known to affect the gelation process. Regarding the polymer concentration, Wu et al. (2017) found that at concentrations lower than 10% and higher than 30%, the viscosity of SLP solutions was extremely high, hampering the injection. Likewise, gelation times at 37 °C were influenced by the initial temperature and the dispersion medium of the samples [20]. They also confirmed that sol-gel transitions were related to the formation and collapse of the hydrogel network. While the viscosity of the sol was undetectable between 25–30 °C at 10% SLP, a rapid rise occurred when the temperature exceeded the LCST (35 °C), pointing out the cross-linkage formation. This was followed by a rapid drop in viscosity and, eventually, the collapse of the gel. From 35 °C to 37 °C, a slight increase in viscosity was observed. Additionally, at higher SLP concentrations (20%), the viscosity remained unaffected by changes in shear rate or temperature [20].

Clinical and non-clinical toxicological data available from the manufacturer confirm the safety of SLP for oral use in adults, even at high doses [16,21,22,23]. It has been tested in multiple clinical trials across various countries (France, Germany, UK, USA, and India) without displaying safety concerns. Toxicity studies in rats determined an LD50 > 5,000 mg/kg, while genotoxicity tests showed no mutagenic effects. Additionally, SLP demonstrated no teratogenic potential or adverse effects on fertility and prenatal development. SLP is non-irritating, non-sensitizing, and does not induce hemolysis at high concentrations [21,22,23].

The amphiphilic nature of SLP that causes its self-aggregation in the form of micelles in aqueous environments permits loading hydrophobic drugs, increasing their solubility and preventing their aggregation and crystallization. Likewise, the hydrophilic PEG segments enhance wettability and dispersibility in biological fluids. SLP may also facilitate the formation of amorphous drug–polymer dispersions, further improving drug dissolution rates [2,24]. These interactions enhance the absorption of drugs in the gastrointestinal tract, promoting their permeation across biological membranes and, ultimately, increasing their bioavailability [24,25].

SLP has been combined with surfactants and polymers to improve the solubility and stability of certain drugs. For instance, the combination of SLP with sodium dodecyl sulfate (SDS) forms a complex that creates supersaturated polymeric micelles for oral delivery of cyclosporine A, which significantly improves its bioavailability. The combination of SLP with surfactants and polymers has potential applications for the delivery of other hydrophobic drugs, particularly under the acidic conditions of the gastrointestinal tract [26].

Similarly, the use of SLP with D-α-tocopheryl polyethylene glycol succinate (TPGS), a surfactant known as an enhancer of drug solubility and permeability, has been explored. This combination has been utilized for increasing the bioavailability of paclitaxel, curcumin, and other anticancer agents [27,28,29]. The formation of mixed micelles between SLP and TPGS enhances the solubilization capacity by combining the amphiphilic properties of both surfactants, offering a versatile platform for drug delivery for both hydrophilic and hydrophobic drugs.

SLP has also been combined with poloxamers, such as Pluronic F127, Pluronic 188, Pluronic 105, and Pluronic F108 [30,31,32,33]. The combination of SLP–poloxamer produced mixed micelles with tailored release profiles due to the distinct hydrophilic–lipophilic balance of each poloxamer and demonstrated improvements in the solubility and controlled release of apigenin, magnolol, and docetaxel, making them beneficial in long-term therapies, such as cancer and neurological disorders [30,31,32,33]. These mixed micelles can provide a multi-functional approach to drug delivery by adjusting the release rate, particle size, and drug-loading capacity, depending on the therapeutic need.

Other combinations include SLP with Solutol^®^ HS15, a non-ionic surfactant, a combination that has been shown to improve the solubilization of hydrophobic drugs such as docetaxel and curcumin [34,35]. The mixed micelles thus formed with these surfactants offer good biocompatibility and reduced toxicity, suitable for parenteral administrations. Additionally, combinations of SLP with sodium cholate and glycyrrhizic acid have been explored in order to enhance drug solubility and gastrointestinal absorption. These formulations are capable of increasing the solubility of poorly water-soluble drugs and forming stable mixed micelles that enhance drug permeability across biological membranes [36,37].

## 3. SLP Formulation Methods

Various methods of formulation have been employed to harness the properties of SLP. These methods, including hot-melt extrusion, spray drying, drug–polymer layering, and capsule and tablet formulations, are designed to enhance drug solubility, stability, and bioavailability. This section provides an overview of these widespread approaches, highlighting their application and benefits in the development of SLP-based pharmaceutical products.

### 3.1. Hot-Melt Extrusion

**Figure 3:**
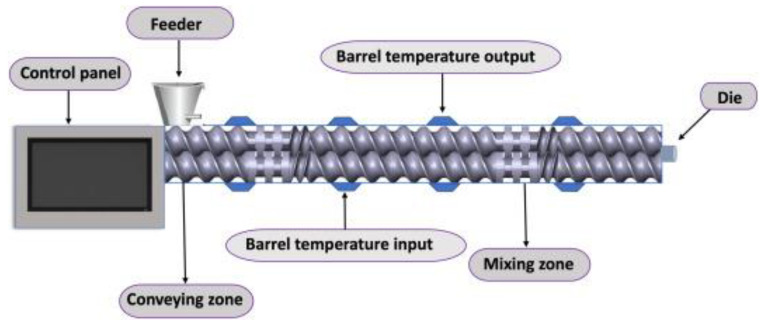
Scheme of the hot-melt extrusion process. From Althobaiti et al., with permission [40].

Hot-melt extrusion (HME) is an effective method for the production of solid dispersion formulations via a solvent-free, fusion-based process. By continuously processing polymeric material under high temperature and pressure (Figure 3), this technique produces uniform solid dispersions that are resistant to shear forces [38]. While traditional HME methods are cost-effective and simple, they present significant drawbacks, such as drug degradation, incomplete miscibility, and potential phase separation during the cooling phase. Therefore, adequate process optimization and careful polymer selection are essential to reduce thermal stress and ensure product stability, particularly for drugs with high melting points [2,39].

SLP is particularly well-suited for HME due to its glass transition temperature of approximately 70 °C, making this polymer easily extrudable with the standard set-up of this technique. For instance, in a typical 16-mm twin-screw extruder, SLP can be processed at temperatures from 120 °C to 220 °C with no signs of chemical degradation. However, the incorporation of drugs leads to a reduction in the extrusion temperature below 120 °C because of drug degradation [16].

Han et al. (2015) prepared solid dispersions of dronedarone hydrochloride, a drug known for its poor aqueous solubility, using SLP, aiming to enhance its pharmacokinetic profile. This study focused on optimizing the formulation parameters, including the SLP/drug ratio and the processing conditions during extrusion. The solid dispersions produced exhibited improvements in drug release profiles compared with the pure drug. Additionally, this study emphasized the advantage of HME in achieving a uniform distribution of dronedarone hydrochloride within the polymer matrix, thus preventing crystallization and ensuring stability [38]. This same goal was pursued by Darwich et al. (2023) as they further explored the potential of HME for the solubility enhancement and release profiles of itraconazole, a poorly soluble antifungal agent. They prepared solid dispersions of the drug with SLP by optimizing the drug-to-polymer ratio and HME processing conditions, observing that the solid dispersions improved the solubility of itraconazole compared with the crystalline form while maintaining the homogeneous distribution of the drug within the SLP matrix [41]. Likewise, Karami et al. (2024) reported the formulation of ibuprofen with the acrylic resin Eudragit^®^, poly(methyl methacrylate-co-methacrylic acid), and SLP at processing temperatures of 150 °C to 160 °C. Their findings revealed that specific ratios of these polymers significantly improved the dispersion and stability of the drug, effectively reducing the crystallinity and enhancing its solubility. They also noticed that Eudragit^®^ and SLP showed thermal compatibility within the studied temperature range, confirming the suitability of HME for the production of stable, homogeneous formulations with minimal degradation risk [42]. Another example of SLP-based formulations via HME is described in the work by Li et al. (2024), in which they investigated the production of amorphous solid dispersions of lumefantrine, an antimalarial drug. By employing a high drug load of lumefantrine, they successfully prepared solid dispersions that exhibited improved solubility compared with the native form of the drug [43].

Another illustration is the work by Winck et al. (2023), in which they optimized the residence time and melt temperature using a blend of SLP and Eudragit^®^ with ibuprofen. This study emphasized the importance of controlling speed, feed rate, and extrusion temperature for maintaining a stable polymer flow in order to achieve the uniform dispersion of ibuprofen within the polymer matrix, thus ensuring the stability and efficacy of the final product. In their experimental set-up, a two-compartment approach to model the residence time distributions was used by imitating the behavior of a pipe and a stirred tank. Shorter residence times at optimized melt temperatures helped in the prevention of thermal degradation of the model drug used, which would be particularly useful for heat-sensitive drugs [44].

Darwich et al. (2024) have explored an original approach to enhance, by making use of HME, the release profiles of poorly soluble ionizable drugs, whose solubility and release characteristics are strongly affected by the pH, especially in the gastrointestinal environment. They formulated ionizable ibuprofen in a polymer matrix of SLP, optimizing the polymer-to-drug ratio and extrusion conditions to further achieve a stable amorphous state with enhanced solubility. The solid dispersion was characterized by X-ray diffraction (XRD) and DSC, and the results indicated that the dispersions maintained an amorphous state, which is a critical factor in achieving a pH-independent release profile. Notably, this study was successful in modulating the drug release rate, which remained constant, independently of the pH range considered [45].

### 3.2. Spray Drying

Spray drying is a widely adopted method for the production of solid dispersions, offering a solvent-based approach that can achieve high drug loads, solubility enhancement, and improved bioavailability of poorly soluble drugs. This technique involves the atomization of a solution containing the drug and excipients into small droplets, followed by rapid solvent evaporation to yield a dry powder. SLP is a particularly suitable polymer for spray drying because of its high solubility in volatile organic solvents, such as methanol, ethanol, and acetone. Its low viscosity in solution further facilitates the processing, enabling efficient spray drying without risking nozzle clogging or uneven particle formation. The concentrations for spray drying typically range from 5% to 30% *w*/*w*, tailored based on factors such as the selection of solvent, solution viscosity, drug concentration, and specific spray dryer conditions [16].

Some recent works have reported on SLP-based formulations that make use of spray drying. Rahman et al. (2023) described a spray drying approach designed to produce hybrid nanocrystal-amorphous solid dispersions in order to improve the supersaturation and solubility of ritonavir, an antiretroviral primarily used to treat AIDS. They described an enhanced solubility and a prolonged supersaturation for this drug, concluding that this technique was able to prevent crystallization [46].

A similar study by Koleva et al. (2024) reported on the spray-dried encapsulation of eugenol, a natural antioxidant and antimicrobial with limited water solubility, using a combination of SLP and Pluronic F127 as carriers. This formulation, aimed at the design of stable and water-dispersible powders suitable for various pharmaceutical and food applications, achieved a high encapsulation efficiency for formulations with lower eugenol concentrations. They also demonstrated that the addition of the poloxamer to the SLP improved the flow properties of the spray-dried powders, although the overall yield was slightly reduced [47].

Bajaj et al. (2024) conducted a similar investigation focusing on the preparation of mebendazole-loaded SLP polymeric micelles to enhance both the solubility and bioavailability of this antiparasitic, poorly water-soluble drug. Spray drying effectively improved the drug solubility and absorption, achieving significantly higher bioavailability compared with its crystalline forms [48].

Kushwah et al. (2024) compared HME and spray drying in terms of drug solubility and dissolution properties by preparing amorphous solid dispersions of BCS class II drugs carvedilol, ibuprofen, and fenofibrate, using SLP and hydroxypropyl methylcellulose (HPMC) matrices. As mentioned above, SLP is suited for both HME and spray drying due to its thermoplastic nature and the variety of solvents in which it can be solubilized. In contrast, HPMC features weaker hydrogen bond acceptors, affecting its ability to stabilize certain drugs in amorphous form. Their results confirmed that HME generally provided more stable amorphous solid dispersions because of the higher degree of interaction between the drug and the polymeric matrix, thus enhancing the drug solubility. On the contrary, spray drying formed amorphous solid dispersions with improved dissolution profiles but with slightly lower stability. Therefore, depending on the desired target for the formulation, both preparation methods should be considered [49]. Spray drying allows high drug loading, greater control over particle characteristics, and suitability for both oral and inhalable formulations [50]. Compared with HME, spray drying is especially advantageous for thermosensitive compounds because of its relatively low-temperature processing.

### 3.3. Electrospinning

**Figure 4:**
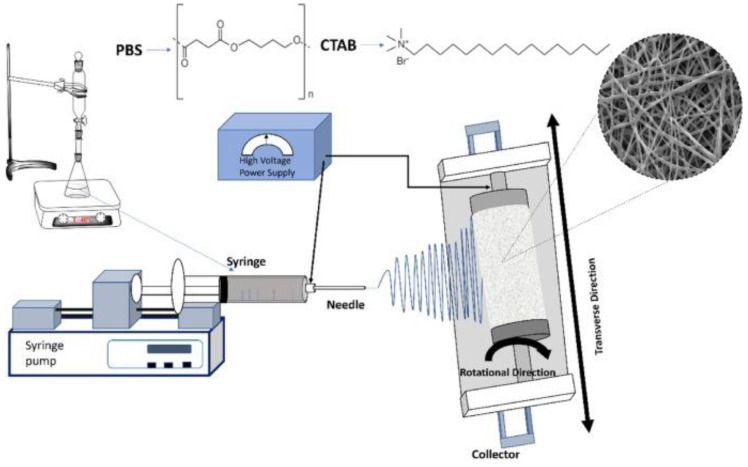
Schematic representation of a solution electrospinning setup. From Bonakdar et al., with permission [57].

Electrospinning is a versatile and widely utilized technique for the manufacturing of micro and nanofibers, structures that have widespread applications, not only in drug delivery but also in tissue engineering and filtration systems, among others (Figure 4) [51,52,53,54,55,56,57]. This process involves applying a high voltage between the collector and the polymer solution, causing the solution to be ejected from a syringe at a constant rate to form fine fibers. The resulting nanofibers have a high surface area-to-volume ratio that benefits the enhancement of the solubility and dissolution rates of poorly soluble drugs [51,56]. The properties of electrospun nanofibers make them particularly advantageous for the formulation of solid dispersions [51]. Drugs can be embedded within polymeric matrices using electrospinning to tune the dissolution profile and improve their stability. Additionally, different polymers can be combined to customize the fiber properties in order to meet specific formulation requirements [58,59].

Recent progresses in electrospinning applications in which SLP is utilized support its potential for creating various morphologies and structures, including aligned fibers and porous mats. For instance, Tipduangta et al. (2021) explored the stability of amorphous solid dispersions by combining SLP and PVP, aiming at preventing crystallization and phase separation. By optimizing the electrospinning conditions, they demonstrated that SLP improved the miscibility when blended with PVP. Moreover, the fibers displayed increased stability and were less prone to crystallization than single-polymer matrices, thus featuring the potential of polymer blending for prolonged storage stability [60]. Pisani et al. (2021) also fabricated nanofibers combining PVP and SLP with the addition of sodium lauryl sulfate (SLS), concluding that this formulation caused a significant increase in the solubility rate of meloxicam [61].

**Figure 5:**
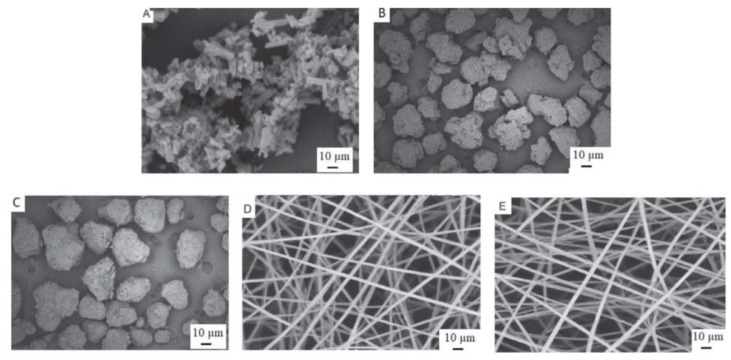
Scanning electron microscope images (**A**) native efavirenz, (**B**) SLP as received, (**C**) physical mixture of efavirenz and SLP, (**D**) efavirenz-loaded SLP nanofibers, and (**E**) SLP nanofibers. From Ahmed et al., with permission [63].

Another study by Gomaa et al. (2022) reported on SLP-based nanoparticles containing griseofulvin via electrospinning. Instead of using a conventional syringe, the electric field generated sufficed to pull the solution from the needle tip, producing nanoparticles, with the associated benefit in equipment requirements. This process yielded small, homogeneous nanoparticles with a nearly six-fold drug dissolution enhancement and improved bioavailability [62]. More recently, Ahmed et al. (2024) prepared electrospun fibers of SLP loaded with efavirenz (Figure 5), a retroviral used for the treatment of HIV-1 infection. The resulting material was able to enhance the dissolution rate and bioavailability of the drug [63].

### 3.4. Drug–Polymer Layering

This technique involves layering a polymer over granules, beads, or other substrates in a fluid bed coater. In this method, the polymer and drug are dissolved in ethanol, methanol, or acetone to achieve a uniform solution before coating. During the process, conditions such as solvent type, concentration, and temperature must be carefully controlled to avoid premature evaporation and ensure an even coating distribution [16,64]. SLP has been employed for layering poorly soluble drugs. In this study by Batra et al. (2021), the authors investigated the effects of SLP and other polymers as binders for layering drug granules using twin-screw melt granulation, a similar process to fluid bed layering, but with melting steps. Specifically, they studied how this polymer’s binding capabilities improved the tableting properties and mechanical strength of granules of two poorly soluble drugs, metformin hydrochloride and acetaminophen. They demonstrated that high concentrations of SLP (at 10% *w*/*w*) improved granule compressibility and uniformity, the concentration being a critical factor for tablet formation [64].

In addition to conventional fluid bed coating, alternative layering methods have been explored to enhance the delivery of poorly soluble drugs through precise drug–polymer layering. Abid et al. (2021) developed an innovative “hot punching” approach for layering budesonide, an anti-inflammatory drug, combined with SLP on biodegradable microcontainers. Their approach involved heating SLP with the drug to create a thin film layer on microscale polymeric containers. The application of hot punching resulted in the formation of a consistent polymer layer encapsulating the drug effectively. Furthermore, they found that this method enhanced budesonide’s solubility and bioavailability, as SLP was able to maintain the drug in an amorphous state [65]. In the same line, Salawi et al. (2021) followed a similar strategy for improving the bioavailability of hydrophobic vitamin E in combination with SLP, suggesting that the ability to form water-compatible coatings could be extended to other hydrophobic drugs [66].

As a last example in this section, it is worth mentioning a study by Fernandez-Garcia et al. (2023), in which SLP was used to coat granules containing the poorly soluble antifungal drugs, amphotericin B and itraconazole, aimed at enhancing their oral bioavailability when administered jointly. Following the drug layering, SLP was applied as an additional coating layer, stabilizing the formulation and promoting enhanced solubility and controlled release of the active agents for oral administration [67].

### 3.5. Capsule Formulation

Capsule formulation is a widely used method in the pharmaceutical industry, particularly for poorly soluble compounds. Due to its self-aggregation properties, SLP can solubilize hydrophobic drugs, which can significantly improve their dissolution rates and bioavailability when administered orally [2]. As mentioned above, SLP is compatible with a variety of solvents and, therefore, can be incorporated into different capsule types, such as hard or soft gelatin-based ones. SLP can thus be incorporated as a solid dispersion or as a component of self-emulsifying drug delivery systems (SEDDS). The ability of SLP to form stable, amorphous solid dispersions helps prevent recrystallization, which is a common issue with poorly soluble drugs [2,68]. Furthermore, the use of SLP in capsules is advantageous because it ensures a more consistent and rapid absorption of the active compound in the gastrointestinal tract [68].

**Figure 6:**
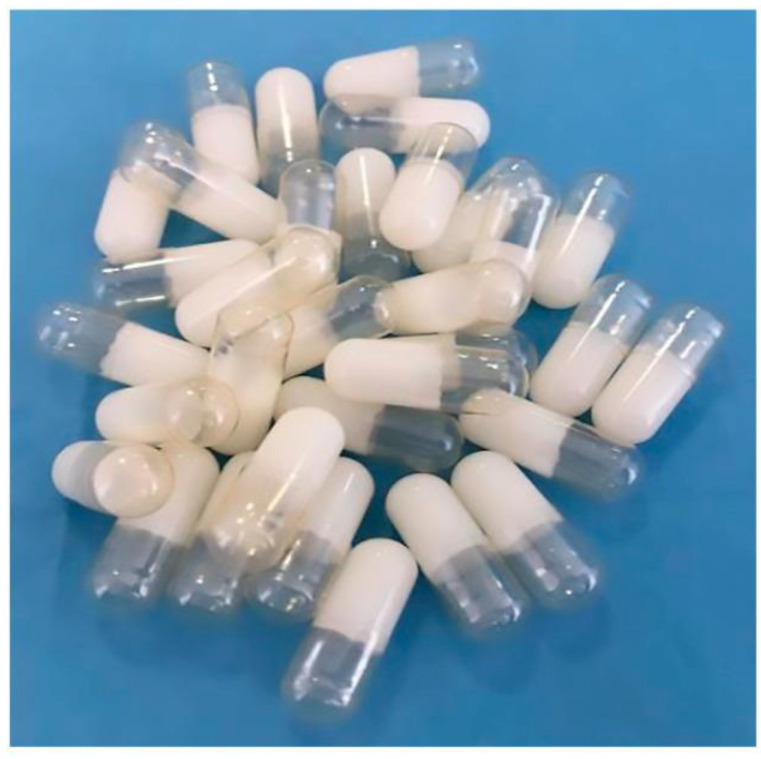
Solidified self-emulsifying drug delivery system (SEDDS) formulation of aprepitant with Soluplus^®^, Capryol^TM^, Kolliphor^®^ CS20 and Transcutol^®^ P in hard gelatin capsules for oral administration. From Nazli et al., with permission [71].

Some recent studies have described the use of SLP for capsule formulations. For instance, Dukhan et al. (2018) investigated formulations of gliclazide, a challenging compound in terms of solubility. They prepared solid dispersions of the drug with SLP in a 1:8 ratio using solvent evaporation. Then, the product was formulated into gelatin capsules, which showed improved dissolution profiles compared with the physical mixture drug–polymer [69]. The same objective was pursued by Desai et al. (2023), who investigated the use of SLP as a carrier aimed at enhancing the oral delivery of arteether, an antimalarial poorly water-soluble drug, in the form of capsules. The formulation showed enhanced solubility and a stable amorphous state, which allowed improved dissolution rates and bioavailability of the drug by oral administration [70]. A similar study was conducted by Nazli et al. (2023), in which they utilized SLP as a polymeric precipitation inhibitor in a semi-solid SEDDS for the oral delivery of aprepitant, a poorly soluble drug used for the blockage of nausea and vomiting. The formulation process involved melting SLP along with other excipients, such as Kollidon^®^ VA64 and PVP, by incorporating the drug at different concentrations. The optimized formulation consisted of 100 mg of Capryol^TM^ 90, 500 mg of Kolliphor^®^ CS20, 400 mg of Transcutol^®^ P, 100 mg of SLP, and 40 mg of aprepitant, which was introduced into gelatin capsules for oral administration (Figure 6). This formulation showed improved solubility, dissolution profiles, and bioavailability compared with the pure aprepitant, as well as that the drug released faster from the optimized formulation than the commercial product [71].

### 3.6. Tablet Formulation

Tablet formulation is one of the most employed dosage forms because of its stability and patient acceptability. This technique allows the drug to be physically and chemically protected, as well as to mask some possible bitter tastes [72,73,74,75,76,77,78].

As mentioned in Section 3.3, Pisani et al. (2021) explored the use of SLP in electrospun nanofiber mats to improve the dissolution rate of meloxicam and then compressed these mats into tablets. The nanofibers increased the surface area of the drug, thus promoting faster dissolution [61].

Browne et al. (2021) utilized SLP for the preparation of solid dispersions of nifedipine, employed for the treatment of high blood pressure and angina pectoris, in which the spray-dried components were then compressed into tablets. They compared the effect of different polymers (SLP, HPMC, and PVP) and found that the former was the most suitable for rapid-dissolving formulations, which, in addition, improved the dissolution of nifedipine [79]. Kanojiya et al. (2022) developed a fast-disintegrating tablet containing artemether, an antimalarial drug, using SLP in the solid dispersion [74]. As a last example in this section, Shamim et al. (2024) employed SLP in a surfactant-assisted wet granulation technique to create matrix tablets of ketoprofen, an anti-inflammatory and antipyretic BCS Class II drug. The authors demonstrated that the matrix based on this block copolymer was able to stabilize the formulation and control the release of ketoprofen [80].

## 4. Applications of SLP in Biomedicine

In the previous section we have described a survey of the recent literature on the use of SLP in pharmaceutical formulations. SLP has demonstrated versatility in delivering diverse drug types, including antitumoral, anti-inflammatory, antimicrobial, and antiparasitic agents. Its biocompatibility and stability prompt its use as a carrier in various drug delivery systems, such as nanoparticles, micelles, and solid dispersions, enabling targeted delivery, sustained release, and reduced side effects. By optimizing drug solubility and enhancing bioavailability, formulations with SLP offer solutions to current challenges in treating complex diseases. In this section, we will describe the use of this polymer in selected biomedical applications. A review of the literature has permitted grouping the most recent investigations in three different fields, namely antitumoral, anti-inflammatory and antimicrobial and antiparasitic applications. Other works that do not fit in this classification have been gathered in Section 4.4.

### 4.1. Antitumoral Applications

The design of new antitumoral drug formulations constitutes a field in which SLP has found applications (Table 1). It is the case of paclitaxel, incorporated into liquid lipid crystalline nanoparticles stabilized with SLP. This formulation enhanced drug-carrier compatibility and enabled controlled release, making it an encouraging approach for solid tumor therapy [81]. Similarly, Hexylselen (CPD-3B), an experimental antitumoral agent, was encapsulated in SLP micelles, significantly improving its solubility and stability. These micelles also improved the in vivo antitumoral efficacy of the drug, which makes the formulation potentially suitable for clinical applications [82].

**Figure 7:**
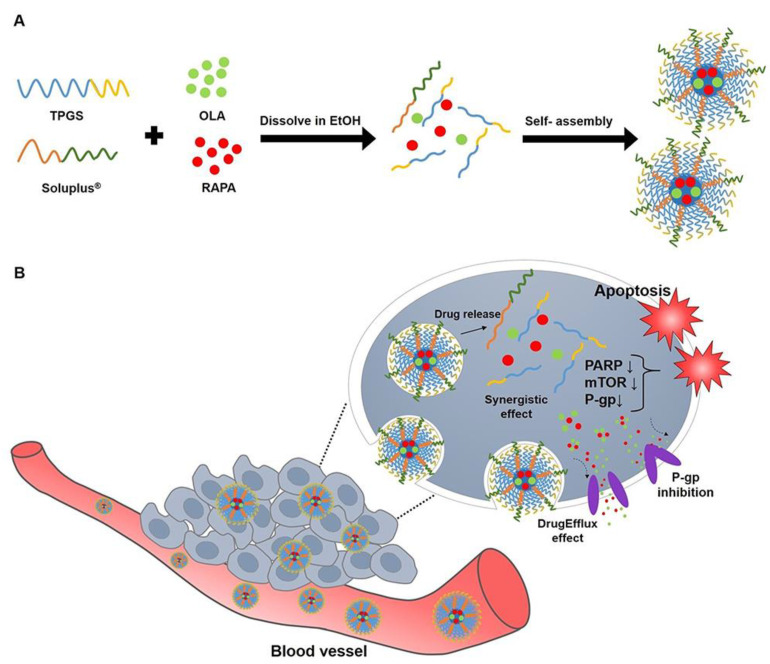
Schematic diagram of (**A**) micelle preparation of TPGS, SOL, OLA, and RAPA and (**B**) representation of the behavior of OLA/RAPA-TPGS/SOL in cancer cells (Abbreviations: TPGS, D-α-tocopheryl polyethylene-glycol (PEG) 1000 succinate; SOL, Soluplus^®^; OLA, olaparib; RAPA, rapamycin; EtOH, ethanol; PARP, poly(ADP-ribose) polymerase; mTOR, mammalian target of rapamycin; P-gp, P-glycoprotein; EPR effect, enhanced permeability and retention). From Shin et al., with permission [87].

Trivedi et al. (2021) formulated *Dioscorea bulbifera* extract in mixed micelles of SLP. This system improved cytotoxicity against cancer cells and showed favorable pharmacokinetics, supporting its use in therapeutic interventions [83]. Taymouri et al. (2021) investigated the incorporation of simvastatin in a nanosuspension of SLP to further encapsulate this system in capsules of Eudragit^®^ and ethyl cellulose. The part of SLP was to solubilize the drug, the formulation aimed at the treatment of colorectal cancer [84]. Similarly, Katona et al. (2022) exploited the surfactant properties of SLP for the fabrication of granules containing megestrol acetate, which is employed in hormone-responsive tumors, such as endometrial or breast cancers [85]. Luteolin, a poorly soluble flavonoid with anticancer properties, was formulated into amorphous solid dispersions with SLP, which contributed to enhancing its pharmacokinetic parameters [86]. Shin et al. (2024) prepared micelles co-loaded with olaparib and rapamycin for ovarian cancer treatment. In their study, SLP proved effective in stabilizing the formulation, which in turn exhibited synergistic anticancer effects (Figure 7) [87]. In another study, β-ionone formulations utilizing SLP improved the solubility of this insoluble compound, enabling its potential use in many types of cancer therapies [88]. Likewise, nanoparticles combining SLP and chitosan with tamoxifen citrate improved the targeting of breast cancer cells, enhancing its therapeutic effect [89].

Hot-melt extrusion techniques have also been used to prepare solid dispersions of curcumin and piperine with SLP. This combination improved the solubility and bioavailability of both compounds, augmenting their antitumoral effects [40]. Vasarri et al. (2022) prepared freeze-dried solid dispersions of SLP, Solutol^®^ HS15, and TPGS to incorporate usnic acid, which resulted in a reduction in the migrastatic effects in neuroblastoma cells and the improvement of both delivery and therapeutic impact of the drug [90]. Electrosprayed nanoparticles containing everolimus, formulated with SLP and polyvinyl alcohol (PVA), achieved better stability and targeting for cancer therapy [91]. These authors also investigated freeze-thawed nanoparticles containing everolimus, prepared with SLP, which demonstrated effective for the delivery of this drug to brain tumors, a challenging application because of the blood–brain barrier [92]. In the field of oncology diagnostics, SLP, alone and combination with TPGS, was used in radiolabeled nanoparticles of ^99m^Tc intended for imaging and therapeutic intervention monitoring in breast and colon cancers [93].

Additional studies to those presented above have been summarized in Table 1. Altogether, the survey highlights the SLP’s capacity to improve antitumoral drug delivery systems, offering solutions to longstanding challenges in solubility, stability, and targeted delivery, which usually constitute critical challenges in oncology.

**Table 1:** Antitumoral applications of SLP-based formulations.

Drug	Formulation Type of SLP	Role of SLP in the Formulation	Disease or Application	Reference
Albendazole and paclitaxel	SLP, TPGS, and folic acid mixed micelles	Helps in the stability and permits the sustained release of the drugs	Ovarian cancer	[94]
β-ionone	SLP matrix	Increases the solubility and bioavailability of the drug	Many forms of cancer	[88]
Betulinic acid	SLP micelles	Increases the solubility and bioavailability of the drug	Breast cancer	[95]
Brigatinib	SLP and TPGS mixed micelles	Increases the solubility and bioavailability of the drug	Lung cancer (non-small cell lung carcinoma, NSCLC)	[96]
Camptothecin analog FLQY2	SLP micelles produced by solvent evaporation	Increases the solubility and bioavailability of the drug	Solid tumors	[97]
Chrysin	SLP and TPGS micelles prepared by solvent evaporation	Increases the solubility and bioavailability of the drug	Hepatocellular carcinoma and anti-inflammatory response	[98]
CPD 23	SLP micelles	Acts as a carrier and enhances the pharmacokinetics of this drug, augmenting its stability in blood	Kidney tumors	[99]
Curcumin and piperine	SLP solid dispersion prepared via HME	Increases the solubility and bioavailability of the drugs	Antitumoral and anti-inflammatory properties	[40]
DHA-S-CA	SLP and TPGS nanomicelles	Increases the solubility and bioavailability of the drug	Lung cancer cells	[100]
*Dioscorea bulbifera* extracts	SLP + poloxamer F127 films produced by thin-film dispersion	Increases the solubility of the drug and enhances cytotoxicity in tumor cells	Apoptosis of tumors	[83]
Docetaxel	SLP and poloxamer F108 micelles produced by spray drying	Helps in the stability and permits the sustained release of the drug	Melanoma	[33]
Docetaxel and curcumin	SLP and TPGS mixed micelles	Permits the sustained release of the drugs	Breast cancer	[101]
Everolimus	Electrosprayed SLP-PVA	Helps in the stability and improves the targeting of the drug	Cancer therapy	[91]
Everolimus	Electrosprayed SLP-PVA	Helps in the stability and improves the targeting of the drug	Brain tumors	[92]
Gemcitabine and vitamin E succinate	SLP micelles	Helps in the stability and enhances the affinity of the drugs	Pancreatic cancer	[102]
Hexyselen (CPD-3B)	SLP micelles	Increases the solubility of the drug	Kidney tumors	[82]
Luteolin	SLP amorphous solid dispersions	Increases the solubility and bioavailability of the drug	Antioxidant, antimicrobial, anti-allergic, cardio-protective, and anti-cancer activities	[86]
Magestrol acetate	SLP and Cremophor^®^ RH 40 mixed micelles	Acts as a carrier and stabilizes the drug	Endometrial and breast cancers and HIV infections	[85]
Naringin	SLP, poloxamer 188, Kollidon^®^ VA30 and Kollidon^®^ VA64 solid dispersions prepared by freeze-drying	Increases the solubility and bioavailability of the drug	Neuroblastoma, antibacterial properties	[103]
Olaparib	SLP matrix prepared by antisolvent precipitation	Helps in the stability of the drug and enhances cytotoxicity in tumor cells	Various types of cancer	[104]
Olaparib and rapamycin	SLP and TPGS mixed micelles	Helps in the stability of the drugs	Ovarian cancer	[87]
Paclitaxel	SLP + poloxamer 407 + Tween 80 stabilizers for lipid nanoparticles	Helps in the stability of the drug	Many types of tumors	[81]
Paclitaxel	Glycosylated-SLP and TPGS matrix	Increases the solubility and bioavailability of the drug	Glioblastoma cell lines	[105]
Paclitaxel	SLP nanomicelles	Increases the solubility of the drug	Breast cancer (triple negative breast cancer, TNBC)	[106]
Paclitaxel and curcumin	SLP and TPGS matrix	Acts as a matrix and carrier for the delivery of these drugs	Breast and ovarian cancers	[107]
Paclitaxel and resveratrol	SLP nanoparticles produced by thin-film hydration	Increases the bioavailability of the drugs	Glioma	[108]
Quercetin	Polymeric mixed micelles of SLP, vitamin E, TPGS, and poloxamer 407	Acts as a matrix	Human U87MG glioma cells	[109]
Quercetin	SLP micelles produced by thin-film hydration	Increases the solubility of the drug and reduces off-target toxic effects	Tumor angiogenesis	[110]
Radiolabeled bevacizumab	SLP micelles and SLP-TPGS mixed micelles	Allows the targeting and imaging	Breast and colon cancers	[93]
Silymarin	SLP, Kollidon^®^ VA64, and poloxamer 188 solid dispersions prepared by solvent evaporation, microwave irradiation, and freeze-drying	Increases the solubility of the drug and enhances cytotoxicity in tumor cells	Lung cancer	[111]
Simvastatin	SLP nanosuspension encapsulated in Eudragit^®^ and ethyl cellulose	Increases the solubility of the drug	Colorectal cancer	[84]
Tamoxifen citrate	SLP and chitosan nanoparticles	Increases the targeting and therapeutic effect	Breast cancer	[89]
Usnic acid	SLP + TPGS + Solutol^®^ HS15 micelles produced by freeze-drying	Reduces cell migration and enhances the stability and activity of the drug	Human SH-SY5Y neuroblastoma cells	[90]

### 4.2. Anti-Inflammatory Applications

Another field in which SLP is finding ample use is in the treatment of inflammation caused by different pathologies. The investigations in this area have aimed at enhancing the solubility, bioavailability, and therapeutic efficacy of anti-inflammatory drugs (Table 2). For example, Pisani et al. (2021) [61] and Wang et al. (2022) [112] have established that SLP improves the dissolution rate of meloxicam and glycyrrhetinic acid, respectively. The common thread between these studies is SLP’s ability to enhance the solubility of the considered drug. As mentioned before, Pisani et al. utilized electrospun nanofibers to optimize the drug release [61], a strategy that is complementary to Wang et al.’s solid dispersion formulation for improving the bioavailability of glycyrrhetinic acid, a compound with strong hepatoprotective and anti-inflammatory effects [112]. Similarly, Pagano et al. (2021) explored a different formulation approach by developing sodium carboxymethyl cellulose and SLP-based hydrogels for the delivery of 18β-glycyrrhetinic acid, the main metabolite of glycyrrhetinic acid, providing a promising strategy for the localized treatment of wounds and inflammation [113]. Likewise, Prasad et al. (2022) produced and tested SLP-based solid dispersions of mefenamic acid in combination with HME, concluding that this formulation stabilized this anti-inflammatory drug [114]. Another study was conducted by Althobaiti et al. (2022), who underscored the role of SLP in enhancing the solubility and therapeutic efficacy of curcumin combined with piperine, resulting to be more effective when formulated with SLP [40].

In a different approach, Sipos et al. (2023) [115] and Shamim et al. (2024) [80] used SLP in formulations aimed at improving the delivery of drugs to targeted sites, namely the central nervous system (CNS) and systemic circulation, respectively. Sipos et al. proved that SLP facilitates the nasal delivery of meloxicam, enhancing its CNS penetration and offering a promising strategy for treating neuroinflammatory conditions [115]. Shamim et al., on the other hand, developed ketoprofen matrix tablets with SLP, which allowed prolonged drug release and enhanced anti-inflammatory effects over time [80]. Interestingly, the work of Chhimwal et al. (2023) focused on targeting the mTOR/SREBP-1c axis to treat non-alcoholic fatty liver disease (NAFLD), a condition with an important inflammatory component. SLP helped improve the bioavailability of phloretin [116]. Lastly, Ashokbhai et al. (2024) utilized additive manufacturing technologies to create 3D-printed etoricoxib tablets, where SLP enhanced the drug’s solubility and controlled release [117].

Table 2 compiles a selection of recent studies on SLP-based formulations of anti-inflammatories for enhanced therapeutic or pharmacokinetic effects.

**Table 2:** Anti-inflammatory applications of SLP-based formulations.

Drug	Formulation Type of SLP	Role of SLP in the Formulation	Disease or Application	Reference
18β-glycyrrhetinic acid	SLP + sodium carboxymethyl cellulose hydrogels	Increases the solubility and permits the sustained release of the drug	Inflammation in wound treatment	[113]
Aloe emodin	SLP and glycyrrhizic acid micelles prepared by thin-film hydration	Increases the solubility and bioavailability of the drug	Gouty arthritis (hyperuricemia)	[37]
Atorvastatin	SLP solid dispersion prepared by a super critical fluid technology	Increases the solubility and bioavailability and permits the sustained release of the drug	Inflammatory Bowel Disease and Irritable Bowel Syndrome	[118]
Budesonide	Microcontainers of polycaprolactone (PCL) coated by SLP films	Permits the amorphous state maintenance and the sustained release of the drug	Inflammatory Bowel Disease	[65]
Chrysin	SLP and TPGS micelles prepared by solvent evaporation	Increases the solubility and bioavailability of the drug	Hepatocellular carcinoma and anti-inflammatory response	[98]
Colchicine	SLP microarray patches	Acts as a matrix and carrier for the delivery of the drug and permits a sustained release	Gout	[119]
Curcumin	Solid dispersion formed with SLP, Syloid^®^, poloxamer 188 and HPMC E5	Increases the solubility and bioavailability of the drug and enhances its activity	Anti-inflammatory and antimicrobial responses	[120]
Curcumin and piperine	SLP solid dispersion prepared via HME	Increases the solubility and bioavailability of the drugs	Antitumoral and anti-inflammatory properties	[40]
Etoricoxib	SLP produced by HME and 3D-printed tablets	Increases the solubility and permits the sustained release of the drug	Anti-inflammatory properties	[117]
Flurbiprofen	Pseudopolyrotaxane preparation upon the mixing of SLP micelles and cyclodextrins	Permits the sustained release of the drug	Anterior uveitis (eye inflammation)	[121]
Glycyrrhetinic acid and L-arginine	SLP solid dispersion	Increases the solubility and bioavailability of the drugs	Anti-inflammatory activity for gastric ulcers	[112]
Ivermectin	SLP microarray patches	Promotes the mechanical robustness and increases the solubility of the drug	Rosacea disease	[122]
Ketoprofen	SLP tablets prepared by wet granulation	Increases the solubility and bioavailability of the drug	Anti-inflammatory properties	[80]
Mefenamic acid	SLP + sorbitol matrix produced via HME	Helps in the stability of the drug	Anti-inflammatory properties	[114]
Meloxicam	SLP and poloxamer F127 prepared by fusion and HME	Increases the solubility of the drug	Anti-inflammatory activity on RAW macrophages	[123]
Meloxicam	Electrospun nanofibers of SLP and SLS for tablet formulations	Increases the solubility of the drug	Anti-inflammatory activity	[61]
Meloxicam	SLP micelles for nasal administration	Promotes the transport to the CNS	Anti-inflammatory activity for brain applications	[115]
Narasin	Self-nanomicellizing solid dispersions of SLP in the form of a gel	Permits the skin penetration of the drug and increases its solubility	Anti-inflammatory response for acne and activity against antimicrobial-resistant strains of *Cutibacterium acnes*	[124]
Phloretin	SLP amorphous solid dispersion	Increases the solubility and bioavailability of the drug	NAFLD	[116]
Pterostilbene	SLP and poloxamer 188 mixed micelles	Increases the solubility and bioavailability of the drug	Anti-inflammatory properties in acetaminophen-induced acute liver injury	[125]

### 4.3. Antimicrobial and Antiparasitic Applications

Recent studies have reported the use of SLP in the development of antimicrobial and antiparasitic formulations, addressing the challenges associated with poorly soluble drugs for infectious and parasitic diseases (Table 3). In fact, Takale et al. (2022) demonstrated that lumefantrine, an antimalarial drug, exhibited improved solubility, bioavailability, and antiparasitic activity when formulated as solid dispersions with SLP, piperine and other excipients, such as hydroxypropyl cellulose (HPC) and Pluronic F68 [126]. In another study, Molina et al. (2022) utilized SLP to microencapsulate emamectin benzoate, a drug used for the treatment of helminthiasis, achieving enhanced dissolution and permeability through fish intestinal membranes [127].

Joshi et al. (2022) studied SLP as a precipitation inhibitor in drug–drug combination therapies. In this formulation, they combined albendazole and mebendazole, antiparasitic agents effective against *Caligus rogercresseyi*, and solubilized both in an SLP matrix. Its ability to maintain supersaturation ensured enhanced bioavailability and reduced drug precipitation for co-administration of these antimicrobials [128]. Moreover, Paulino et al. (2023) [129] and Rocha et al. (2023) [130] developed novel schistosomicidal formulations using SLP-based solid dispersions containing PVP K-30 and PEG as excipients. Both studies showed that the polymer matrix was suitable for improving the bioactivity of these hydrophobic experimental drugs, thus providing an effective approach for the treatment of *Schistosoma* infections. In another study, Zhang et al. (2024) investigated the use of carbonitrile derivatives formulated in SLP and hydroxypropyl-β-cyclodextrin solid dispersions to target alternative oxidase inhibitors of *Cryptosporidium parvum*, resulting in an enhancement of the pharmacokinetic parameters of this type of drugs with cryptosporidicidal activity [131].

**Figure 8:**
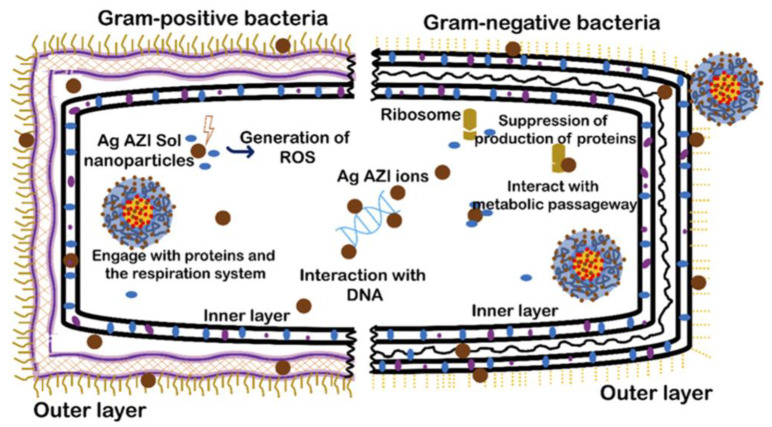
Interaction of Ag-AZI-Sol NPs with Gram-positive and Gram-negative bacteria cell membranes. From Jaligam et al., with permission [132].

Regarding antibacterial applications, Jaligam et al. (2024) synthesized silver-decorated azithromycin nanoparticles with SLP, demonstrating sustained release and enhanced antibacterial efficacy against *Escherichia coli* and *Staphylococcus epidermidis* biofilms (Figure 8) [132]. Likewise, Sipos et al. (2024) explored the combination of SLP and TPGS micelles embedded within a poloxamer 407 gel aiming at the efficient delivery of dexamethasone and tobramycin, with antimicrobial actions through the nasal route for the treatment of nasal rhinosinusitis [133].

Relevant studies, including those aforementioned, encompassing an overview of the most recent advances in the development of effective SLP-based formulations for the treatment of infectious diseases, have been summarized in Table 3.

**Table 3:** Antimicrobial and antiparasitic applications of SLP-based formulations.

Drug	Formulation Type of SLP	Role of SLP in the Formulation	Disease or Application	Reference
Albendazole and mebendazole	SLP matrix	Prevents drug precipitation	Helminthiasis	[128]
Arteether	SLP capsules	Increases the solubility and bioavailability of the drug	Malaria	[70]
*Buddleja globosa* Hope extracts	Spray-dried SLP or PVP	Increases the solubility of the drug and produces enhanced antimicrobial properties	Skin and gastric ulcers, as it has activity against *Pseudomonas aeruginosa*	[134]
Carbonitrile derivatives (LN002)	SLP and hydroxypropyl-β-cyclodextrin solid dispersions	Increases the solubility and bioavailability of the drug	Activity against *Cryptosporidium*	[131]
Carbothioamide derivatives (LQIT/LT-50)	Solid dispersions made of SLP, PVP K-30, and PEG	Increases the solubility of the drug and enhances its activity	Schistosomiasis	[130]
Ciprofloxacin	SLP, PVA, and PEG films prepared by solvent casting	Allows effective drug delivery to the eye and improves corneal and conjunctival permeation	Eye infection	[135]
Curcumin	Solid dispersion formed with SLP, Syloid^®^, poloxamer 188 and HPMC E5	Increases the solubility and bioavailability of the drug and enhances its activity	Anti-inflammatory and antimicrobial responses	[120]
Decoquinate	SLP nanoparticles prepared by HME	Increases the solubility and bioavailability of the drug	Malaria	[136]
Dexamethasone and tobramycin	SLP and TPGS micelles embedded in a poloxamer 407 gel for intranasal administration	Enhances the kinetics of dexamethasone and permits a sustained release of tobramycin	Nasal rhinosinusitis	[133]
Emamectin benzoate	Spray-dried SLP or sodium alginate prepared by ionic gelation	Increases the solubility of the drug, although the best results were obtained with the sodium alginate solid dispersion	Antiparasitic activity against *Caligus rogercresseyi*	[127]
Lumefantrine	SLP, HPC, and poloxamer F68 matrix combined with piperine	Increases the solubility and bioavailability of the drug	Malaria	[126]
Luteolin	SLP amorphous solid dispersions	Increases the solubility and bioavailability of the drug	Antioxidant, antimicrobial, anti-allergic, cardio-protective, and anti-cancer activities	[86]
Mebendazole	Spray-dried SLP micelles	Increases the solubility and bioavailability of the drug and enhances its bioabsorption	Infection from roundworms (pinworms and hookworms), trichinosis, capillariasis, and toxocariasis	[48]
Metronidazole	Microarray patches of SLP	Augments skin permeation of the drug	Skin and soft tissue infections, as it has activity against *Bacteroides fragilus*	[137]
Narasin	Self-nanomicellizing solid dispersions of SLP in the form of a gel	Permits the skin penetration of the drug and increases its solubility	Anti-inflammatory response for acne and activity against antimicrobial-resistant strains of *Cutibacterium acnes*	[124]
Naringin	SLP, poloxamer 188, Kollidon^®^ VA30 and Kollidon^®^ VA64 solid dispersions prepared by freeze-drying	Increases the solubility and bioavailability of the drug	Neuroblastoma, antibacterial properties	[103]
Rifampicin and curcumin	SLP nanomicelles	Allows the drug delivery for an inhalable formulation	Tuberculosis	[138]
Silver-decorated azithromycin	SLP nanoparticles developed by controlled emulsion diffusion	Permits the sustained release of the drug for continuous antibacterial efficacy	Antibacterial efficacy against *Escherichia coli* and *Staphylococcus epidermidis*	[132]
Thiazolidine derivatives (LPSF/GQ-238)	Solid dispersions made of SLP, PVP K-30, and PEG	Increases the solubility of the drug	Schistosomiasis	[129]

### 4.4. Other Biomedical Applications

This section is dedicated to recent investigations that make use of SLP, emphasizing the role of the copolymer in advancing treatment strategies (Table 4).

One noteworthy application involves the interaction of SLP nanomicelles with protein coronas, as reported by Wang et al. (2022) [139], aimed at the role as a nanocarrier of this polymer that derives from its capacity of enhancing the stability and biodistribution of encapsulated drugs. Another application encompasses the cytokine-free expansion of human hematopoietic stem cells (HSCs), as reported by Sakurai et al. (2023). While not directly a formulation component, SLP biocompatibility allows the HSC expansion, which could help in the development of therapies for blood-related disorders [140]. Recently, Karami et al. (2024) utilized a core–shell floating tablet of ketamine hydrochloride fabricated via 3D printing. SLP enabled precise control over drug release, ensuring consistent plasma levels for the management of refractory depression and chronic pain [42]. In another study, Paczkowska-Walendowska et al. (2023) employed SLP in HME formulations of polydatin derived from *Polygonum cuspidatum*. This strategy enhanced the physicochemical properties of the extract, supporting its use in buccal applications [141].

Regarding regenerative medicine applications, Al-Sudani et al. (2024) investigated SLP/keratin-based nanofibers loaded with merwinite nanoparticles and sildenafil for vascular–osteogenic regeneration [142]. In the formulation devised, SLP achieved uniform drug distribution and improved the bioactivity of the drug. This original approach may have consequential implications for bone tissue engineering, addressing critical needs in the treatment of osteogenic defects [142].

A summary of this section with selected applications is presented in Table 4. The studies surveyed in this section shed light on the SLP capacities and their growing importance in modern drug delivery systems, illustrating the broad spectrum of SLP applications that may pave the way for future research in enhancing therapeutic efficacy.

**Table 4:** Other applications of SLP-based formulations.

Drug	Formulation Type of SLP	Role of SLP in the Formulation	Disease or Application	Reference
Agomelatine	Intranasal gel containing SLP, hydroxypropyl-β-cyclodextrin and poloxamer 188	Enhances the drug efficacy	Depression	[143]
Bortezomib and lenalidomide	SLP solutions	Enhances HSCs growth	Modification of the culture medium for transplantations	[144]
Carvedilol and curcumin	SLP micelles	Increases the solubility and optimizes the therapeutic potential of the drug	Hypertension	[145]
Curcumin	SLP micelles produced by thin-film hydration	Increases the solubility and bioavailability of the drug	Alcohol-use disorders	[146]
Ketamine hydrochloride	SLP and Eudragit^®^ prepared via HME and then formulated in tablets	Permits the sustained release of the drug	Refractory depression and chronic pain	[42]
Polydatin (*Polygoni cuspidati* extracts)	SLP solid dispersion prepared by HME and then formulated into tablets with HPMC	Increases the muco-adhesivity and enhances the kinetics of the drug	Buccal applications	[141]
Quercetin	SLP microarray patches	Increases the solubility and bioavailability of the drug	Fibrosis lowering, scar formation limitation, and fibroblast proliferation	[147]
Sildenafil	SLP, keratin, and merwinite scaffolds formed via electrospinning	Enhances the osteogenic and angiogenic capacities and allows a robust structure	Bone tissue regeneration	[142]
Tacrolimus	Eye drop formulation formed by Zein-SLP nanoparticles and hydroxypropyl-β-cyclodextrin	Increases the solubility and bioavailability of the drug	Retinal diseases	[148]
No drug was used in this study	SLP nanomicelles	Allows the encapsulation of proteins. Some model proteins employed were bovine serum albumin (BSA), lysozyme, and bovine hemoglobin (BHb)	Allows the encapsulation of drugs for their delivery	[139]
No drug was used in this study	SLP solutions	Permits the long-term ex vivo expansion of HSCs	Hematological diseases	[140]

## 5. Conclusions

SLP is proving to be a trending excipient in pharmaceutical development that offers versatile solutions able to overcome the challenges associated with poorly soluble drugs. SLP’s physicochemical properties permit the stabilization of amorphous drug states, the formation of micelles, the improvement of the pharmacokinetics parameters, and the controlled release of the drug. The diversity of formulations in which SLP can enter facilitates its use for a number of applications in areas that include antitumoral, anti-inflammatory, antimicrobial, and antiparasitic treatments, as well as in emerging biomedical applications such as tissue engineering and diagnostic systems. As research continues to explore the potential of SLP, its integration into personalized medicine, targeted therapies, neglected infectious disease treatments, and advanced manufacturing methods is expected to contribute to pharmaceutical development and the biomedical sciences. This review will be helpful for future studies to expand the understanding and utilization of SLP in the quest for innovative and effective drug delivery systems.
